# Joint analysis of genetic and epigenetic data using a conditional autoregressive model

**DOI:** 10.1186/s12863-018-0641-8

**Published:** 2018-09-17

**Authors:** Xiaoxi Shen, Qing Lu

**Affiliations:** 1 Department of Statistics and Probability, Michigan State University, 619 Red Cedar Rd, East Lansing, MI 48824 USA; 2 Department of Epidemiology and Biostatistics, Michigan State University, 909 Fee Rd, East Lansing, MI 48824 USA

**Keywords:** Joint associations, Conditional autoregressive (CAR) model, Linear score test

## Abstract

**Background:**

Rapidly evolving high-throughput technology has made it cost-effective to collect multilevel omic data in clinical and biological studies. Different types of omic data collected from these studies provide both shared and complementary information, and can be integrated into association analysis to enhance the power of identifying novel disease-associated biomarkers. To model the joint effect of genetic markers and DNA methylation on the phenotype of interest, we propose a joint conditional autoregressive (JCAR) model. A linear score test is used for hypothesis testing and the corresponding *p* value can be obtained using the Davies method.

**Results:**

The JCAR model was applied to the GAW20 data from the Genetics of Lipid Lowering Drugs and Diet Network (GOLDN) study. In our application of the JCAR model, we consider a baseline model and a full model. In the baseline model, we consider 3 different scenarios: a model with only genetic information, a model with only DNA methylation information at visit 2, and a model using both genetic and DNA methylation information at visit 2. For the full model, we consider both genetic and DNA methylation information at visit 2 and visit 4. The top 10 significant genes are reported for each model. Based on the results, we found that the gene *MYO3B* was significant as long as the methylation information was considered in the analysis.

**Conclusions:**

JCAR is a useful tool for joint association analysis of genetic and epigenetic data. It is easy to implement and is computationally efficient. It can also be extended to analyze other types of omic data.

## Background

Advances in high-throughput technologies provide comprehensive assessment of biomarkers, which enable us to systematically study the role of different types of omic data (eg, DNA, DNA methylation, proteins, and metabolites) in human diseases. The collection of multilevel omic data from these studies provides us a great opportunity to integrate information from different levels of omic data into association analysis. Although omic-based association analysis holds great promise for discovering novel disease-associated biomarkers, there is lack of appropriate statistical tools to analyze multilevel omic data [1, 2]. The development of advanced methods to address analytical challenges faced by ongoing omic data analysis can enhance our ability to identify new disease-associated biomarkers.

Many statistical methods have been proposed to study the associations between single-nucleotide polymorphism (SNPs) and disease phenotypes. Although the conventional regression methods (eg, simple linear regression) are easy to use, they are not designed for high-dimensional genetic data analysis, especially with additional omic data (eg, DNA methylation data). Similarity based methods, such as sequence kernel association test (SKAT) [3] or genetic random field model (GenRF) [4], on the other hand, use kernels to construct genetic similarities between individuals, making them applicable for high-dimensional data analysis. Based on the similar idea, we developed a conditional autoregressive (CAR) model for association analysis of sequencing data considering genetic heterogeneity. In this paper, we extend the CAR model for joint association analysis of SNPs and DNA methylation markers. The proposed joint conditional autoregressive (JCAR) model is developed based on a linear mixed model framework by considering the effects of SNPs and DNA methylations, as random effects. A linear score test is then used to perform the association testing.

## Methods

If we are interested in evaluating the association of *K* SNPs and *L* DNA methylation markers in a genetic region (eg, a gene) with a continuous phenotype. A CAR model [5] can be written as the following linear mixed model:

$$ {y}_i={\boldsymbol{x}}_i^T\boldsymbol{\beta} +{g}_i+{m}_i+{\varepsilon}_i,i=1,\cdots, n $$

$$ {g}_i\mid {g}_{-i}\sim \mathcal{N}\left(\frac{\gamma_1}{\sum \limits_{j\ne i}{s}_{ij}^{(1)}}\sum \limits_{j\ne i}{s}_{ij}^{(1)}{g}_j,\frac{\sigma_g^2}{\sum \limits_{j\ne i}{s}_{ij}^{(1)}}\right) $$

$$ {m}_i\mid {m}_{-i}\sim \mathcal{N}\left(\frac{\gamma_2}{\sum \limits_{j\ne i}{s}_{ij}^{(2)}}\sum \limits_{j\ne i}{s}_{ij}^{(2)}{m}_j,\frac{\sigma_m^2}{\sum \limits_{j\ne i}{s}_{ij}^{(2)}}\right) $$

$$ \left[\begin{array}{c}{\varepsilon}_1\\ {}\vdots \\ {}{\varepsilon}_n\end{array}\right]\sim \mathcal{N}\left(\mathbf{0},{\sigma}^2\boldsymbol{K}\right) $$

where *y*_*i*_ is the phenotype of the *i*th subject; ***x***_*i*_ is a *p* × 1 vector of covariates (eg, age, gender, etc.); ***β*** is the fixed effect of the covariates; *g*_*i*_ is the genetic random effect; *m*_*i*_ is the methylation random effect of the *i*th subject; and *ε*_*i*_ is the random error. We can use kinship coefficient matrix ***K*** to model the familial correlations among family members, and the identity matrix ***I*** when samples are independent. ${s}_{ij}^{(1)}$ and ${s}_{ij}^{(2)}$ measure the similarity of the genetic profiles and the similarity of DNA methylation profiles between the *i*th subject and the *j*th subject respectively. *γ*_1_ and *γ*_2_ measure the overall genetic correlation and the overall DNA methylation correlation, respectively.

To test the genetic-only or the methylation-only effect, it suffices to test ${H}_0:{\sigma}_g^2=0$ for genetic effect or to test ${H}_0:{\sigma}_m^2=0$. To evaluate the joint effect of SNPs and DNA methylation markers on the response, we can test the null hypothesis ${H}_0:{\sigma}_g^2={\sigma}_m^2=0$.

A linear score test [6] based on profiled restricted likelihood can then be formed for the association testing. The corresponding score test statistic is

$$ S=\frac{S_1+{S}_2}{2} $$

where

$$ {S}_l=\frac{n-\operatorname{rank}\left(\boldsymbol{X}\right)}{2}\frac{{\boldsymbol{y}}^{\ast^T}\boldsymbol{A}{\boldsymbol{K}}^{-\frac{1}{2}}{\left({\boldsymbol{D}}_l-{\gamma}_l{\boldsymbol{S}}_l\right)}^{-1}{\boldsymbol{K}}^{-\frac{1}{2}}{\boldsymbol{A}}^T{\boldsymbol{y}}^{\ast }}{{\boldsymbol{y}}^{\ast^T}{\boldsymbol{y}}^{\ast }}-\frac{1}{2}\mathrm{tr}\left({\boldsymbol{K}}^{-\frac{1}{2}}{\left({\boldsymbol{D}}_l-{\gamma}_l{\boldsymbol{S}}_l\right)}^{-1}{\boldsymbol{K}}^{-\frac{1}{2}}\right),l=1,2 $$

***A*** is a matrix satisfying $\boldsymbol{A}{\boldsymbol{K}}^{-\frac{1}{2}}\boldsymbol{X}=\mathbf{0}$ and ***AA***^*T*^ = ***I***_*n* − rank(***X***)_ and ${\boldsymbol{y}}^{\ast }=\boldsymbol{A}{\boldsymbol{K}}^{-\frac{1}{2}}\boldsymbol{y}$. ${\boldsymbol{S}}_l=\left[{s}_{ij}^{(l)}\right]$ is the similarity matrix with diagonal elements being 0 and ***D***_*l*_ is a diagonal matrix with the diagonal elements being the row sums of ***S***_*l*_.

The *p* value of the association test can be calculated by

$$ \mathbb{P}\left[{\boldsymbol{y}}^{\ast^T}\boldsymbol{B}{\boldsymbol{y}}^{\ast }>0\right] $$

where

$$ \boldsymbol{B}=\boldsymbol{A}{\boldsymbol{K}}^{-\frac{1}{2}}\left[{\left({\boldsymbol{D}}_1-{\gamma}_1{\boldsymbol{S}}_1\right)}^{-1}+{\left({\boldsymbol{D}}_2-{\gamma}_2{\boldsymbol{S}}_2\right)}^{-1}\right]{\boldsymbol{K}}^{-\frac{1}{2}}{\boldsymbol{A}}^T-\left(\frac{4{S}_l}{n-\operatorname{rank}\left(\boldsymbol{X}\right)}+\frac{1}{n-\operatorname{rank}\left(\boldsymbol{X}\right)}\mathrm{tr}\left[{\boldsymbol{K}}^{-\frac{1}{2}}\left[{\left({\boldsymbol{D}}_1-{\gamma}_1{\boldsymbol{S}}_1\right)}^{-1}+{\left({\boldsymbol{D}}_2-{\gamma}_2{\boldsymbol{S}}_2\right)}^{-1}\right]{\boldsymbol{K}}^{-\frac{1}{2}}\right]\right){\boldsymbol{I}}_{n-\operatorname{rank}\left(\boldsymbol{X}\right)} $$

The *p* value can be calculated using the Davies method [7].

## Results

We conducted a genome-wide gene-based association analysis by applying the new method to genome-wide genetic and methylation data from the Genetics of Lipid Lowering Drugs and Diet Network (GOLDN) study [8]. For the gene-based association analysis, we first extracted SNPs and DNA methylation markers for each gene. There are 13,722 genes with both genetic and DNA methylation information. We started with a baseline model to assess the joint association of genetic and DNA methylation with triglycerides. For this model, we include 717 individuals from visit 2, who have both genetic and DNA methylation information. To evaluate the contribution of SNPs and methylation change to the triglycerides change between visit 2 and visit 4, we fit a full model with 429 subjects who have both genetic and DNA methylation information from visit 2 and visit 4. For individuals with missing genotypes or DNA methylation values, we impute the missing values with the variable mean. We then apply JCAR to the genetic and DNA methylation data, evaluating the potential association of 13,722 genes with triglycerides. In the association analysis, we use the theoretical kinship coefficient matrix to account for familiar correlation among subjects, and adjust for age, gender, and field center.

We considered 3 different analytical strategies for the baseline model (ie, based on visit 2):

1. Genetic information only. In this case, the CAR model can be simplified as

$$ {y}_i={\boldsymbol{x}}_i^T\boldsymbol{\beta} +{g}_i+{\varepsilon}_i,i=1,\cdots, n. $$

The phenotype is the measurements of triglycerides at visit 2 with a normal quantile transformation.

2. DNA methylation information only. In this case, the CAR model can be simplified as

$$ {y}_i={\boldsymbol{x}}_i^T\boldsymbol{\beta} +{m}_i+{\varepsilon}_i,i=1,\cdots, n. $$

The phenotype is the measurements of triglycerides at visit 2 with a normal quantile transformation.

3. Both genetic and DNA methylation information. In this case, the phenotype is the measurements of triglycerides at visit 2 with a normal quantile transformation.

For the full model, *m*_*i*_ is the methylation difference of cytosine-phosphate-guanine (CpG) sites between the 2 visits and the response is the difference of triglycerides at visit 2 and at visit 4 with a normal quantile transformation.

For SNP data, we use the normalized identity-by-state (IBS) kernel as the measurement of similarity; that is,

$$ {s}_{ij}^{(1)}=\sum \limits_{k=1}^K\frac{2-\left|{g}_{i,k}-{g}_{j,k}\right|}{2K} $$

where *g*_*i*, *k*_ and *g*_*j*, *k*_ are, respectively, the genotypes at the *k*th locus for the *i*th and the *j*th subjects and *K* is the total number of SNPs. For DNA methylation data, a Gaussian kernel is used to measure the similarity; that is,

$$ {s}_{ij}^{(2)}=\exp \left\{-\frac{1}{2{\sigma}^2}\sum \limits_{l=1}^L{\left({m}_{i,l}-{m}_{j,l}\right)}^2\right\} $$

where *m*_*i*, *l*_ and *m*_*j*, *l*_ are, respectively, the DNA methylation measurements of the *l*th CpG site for the *i*th and the *j*th subjects. For simplicity, the tuning parameter *σ* is chosen to be the standard deviation of the methylation data. When applying our method to the data, *γ*_1_ is fixed at the average of the entries in the correlation matrix of SNP data, and *γ*_2_ is fixed at the average of the entries in the correlation matrix of DNA methylation data. Tables 1, 2, 3 and 4 summarize the top 10 significant genes. As observed from the 4 tables, no association reached statistical significance after adjusting for multiple comparisons. Although most top 10 significant genes are different for different models, 1 gene, *MYO3B*, is captured by both the baseline model and the full model as long as the methylation information is considered. Further investigation is needed to verify the association and investigate the potential role of *MYO3B* in triglycerides.

**Table 1:** Top 10 significant genes obtained from the baseline model, considering only the genetic information

Gene	Chromosome	*p* Value
*LRIG3*	12	0.000192
*SH3GL1*	19	0.000445
*FBXO17*	19	0.000565
*ETF1*	5	0.000597
*PIF1*	15	0.000676
*GREM1*	15	0.000719
*LEF1*	4	0.000755
*SSTR4*	20	0.000788
*LYZL1*	10	0.000847
*RAB23*	6	0.000903

**Table 2:** Top 10 significant genes obtained from the baseline model, considering only the DNA methylation information

Gene	Chromosome	*p* Value
*MYO3B\*	2	0.000755
*MUCL1*	12	0.001979
*FGFR1OP*	6	0.003126
*IL22RA1*	1	0.003383
*COMMD10*	5	0.003626
*SNX5*	20	0.003696
*DCTN6*	8	0.004189
*KCTD2*	17	0.005595
*CDH4*	20	0.008107
*RWDD3*	1	0.008165

**Table 3:** Top 10 significant genes obtained from the baseline model, considering both  the genetic and DNA methylation information

Gene	Chromosome	*p* Value
*TP53BP1*	15	0.000654
*MYO3B*	2	0.000759
*PLEKHM1*	17	0.000899
*C7orf42*	7	0.000927
*MUCL1*	12	0.00127
*HYAL4*	7	0.001643
*EXOSC10*	1	0.002679
*FGFR1OP*	6	0.002693
*IL22RA1*	1	0.003399
*TP53BP1*	15	0.000654

**Table 4:** Top 10 significant genes obtained from the full model, considering both the genetic and DNA methylation information

Gene	Chromosome	*p* Value
*CYP4A22*	1	0.002192
*MYO3B*	2	0.002254
*C1orf141*	1	0.002534
*C22orf24*	22	0.003132
*SPRR1B*	1	0.005632
*LOC100128076*	9	0.005733
*IKZF2*	2	0.006704
*RANBP6*	9	0.007358
*OR2M2*	1	0.007383
*KLHL29*	2	0.007572

## Discussion

In the application of the JCAR model to the real data, *γ*_1_ and *γ*_2_ are fixed at some value obtained from the SNP and methylation data, respectively. In practice, we do not know the value of *γ*_1_ and *γ*_2_. Therefore, the effect of different values of *γ*_1_ and *γ*_2_ on the results needs further investigation. Similarly, different choices of *σ*^2^ in the Gaussian kernel might also affect the association test, which worths further investigation.

## Conclusions

A JCAR model is proposed for association analysis of genetic data and DNA methylation data. Under the linear mixed model framework, the CAR model is easy to implement and computationally efficient. Although we illustrate the method using the genetic and DNA methylation data, it can be used to analyze other types of omic data (eg, gene expression data) and is capable of analyzing more than 2 levels of omic data. The JCAR model introduced in this paper does not consider the interactions among different levels of omic data. Further study is required to extend the current framework to consider the interactions.

## Abbreviations

CAR: Conditional auto-regression
CpG: Citosine-phosphate-guanine
DNA: Deoxyribonucleic acid
GAW: Genetic Analysis Workshop
GenRF: Genetic random field
GOLDN: Genetics of Lipid Lowering Drugs and Diet Network
JCAR: Joint conditional auto-regression
SKAT: Sequence kernel association test
SNP: Single nucleotide polymorphism
